# Reduced sensitivity to situational change in individuals with autism spectrum condition

**DOI:** 10.1192/j.eurpsy.2021.2033

**Published:** 2021-08-13

**Authors:** S. Tei, J. Fujino

**Affiliations:** 1 Department Of Psychiatry, Kyoto University, Kyoto, Japan; 2 Institute Of Applied Brain Sciences, Waseda University, Saitama, Japan; 3 School Of Human And Social Sciences, Tokyo International University, Saitama, Japan; 4 Medical Institute Of Developmental Disabilities Research, Showa University, Tokyo, Japan; 5 Department Of Psychiatry And Behavioral Sciences, Tokyo Medical and Dental University, Tokyo, Japan

**Keywords:** flexibility, autism, ultimatum game, Attention

## Abstract

**Introduction:**

Individuals with autism spectrum condition (ASC) frequently report difficulties in detecting changes in social situations, which considerably hinder interpersonal communications.

**Objectives:**

To better understand the features of detecting changes in social situations among individuals with ASC.

**Methods:**

Individuals with ASC (N=24) and typical development (TD) (N=24) were included. To examine participants’ sensitivity to situational contexts, we conducted an economic-game task: a modified computer version of the ultimatum game (mod-UG). In UG, two players were offered a chance to win 10 coins after dividing it amongst themselves. The proposer suggests how to split the sum and the responder can accept or reject the deal. After practice, all participants played the role of responders with an imaginary proposer. Participants had to decide whether to accept or reject proposers’ fair/unfair offers. In our mod-UG, additional condition was included that involved intentionality considerations: Unfair offers were displayed with another identical unfair offer. This emphasized the proposers’ inevitable situation of unfair offers. Subsequently, we conducted a 2×2 repeated-measures ANOVA (unfair offers with/without additional cues)×(ASC/TD).

**Results:**

Participants indeed accepted unfair offers significantly more frequently when the other player’s unfair proposal was unavoidable in cue-added conditions, when compared to unfair offers in no-cue conditions. This suggested that participants considered their opponent’s perspective more attentively in cue-added conditions. However, this effect was significantly decreased in the ASC-group (p<0.05; group-condition interaction).

**Conclusions:**

Decreased sensitivity to situational changes among ASC-individuals may be partly due to diminished or inflexible shifting of perspective. Whether this systematized decision-making associates with attentional-bias and stereotyped-behaviors requires further investigation.

**Disclosure:**

No significant relationships.

